# Genome-Wide Identification of Bcl11b Gene Targets Reveals Role in Brain-Derived Neurotrophic Factor Signaling

**DOI:** 10.1371/journal.pone.0023691

**Published:** 2011-09-01

**Authors:** Bin Tang, Pietro Di Lena, Lana Schaffer, Steven R. Head, Pierre Baldi, Elizabeth A. Thomas

**Affiliations:** 1 Department of Molecular Biology, The Scripps Research Institute, La Jolla, California, United States of America; 2 Department of Computer Science and Institute for Genomics and Bioinformatics, University of California, Irvine, Irvine, California, United States of America; 3 Department of Shared Research Services, The Scripps Research Institute, La Jolla, California, United States of America; The University of Hong Kong, Hong Kong

## Abstract

B-cell leukemia/lymphoma 11B (Bcl11b) is a transcription factor showing predominant expression in the striatum. To date, there are no known gene targets of Bcl11b in the nervous system. Here, we define targets for Bcl11b in striatal cells by performing chromatin immunoprecipitation followed by high-throughput sequencing (ChIP-seq) in combination with genome-wide expression profiling. Transcriptome-wide analysis revealed that 694 genes were significantly altered in striatal cells over-expressing Bcl11b, including genes showing striatal-enriched expression similar to Bcl11b. ChIP-seq analysis demonstrated that Bcl11b bound a mixture of coding and non-coding sequences that were within 10 kb of the transcription start site of an annotated gene. Integrating all ChIP-seq hits with the microarray expression data, 248 direct targets of Bcl11b were identified. Functional analysis on the integrated gene target list identified several zinc-finger encoding genes as Bcl11b targets, and further revealed a significant association of Bcl11b to brain-derived neurotrophic factor/neurotrophin signaling. Analysis of ChIP-seq binding regions revealed significant consensus DNA binding motifs for Bcl11b. These data implicate Bcl11b as a novel regulator of the BDNF signaling pathway, which is disrupted in many neurological disorders. Specific targeting of the Bcl11b-DNA interaction could represent a novel therapeutic approach to lowering BDNF signaling specifically in striatal cells.

## Introduction

The striatum plays crucial roles in the planning and modulation of movement and motor function, stereotyped behavior and the establishment of habits [1]–[3], as well as being associated with cognitive function and motivation-related behavior [4]. Precise regulation of gene expression in the striatum is essential to these functions; accordingly, disruption of this regulation can have detrimental consequences, resulting in the development of severe movement disorders and psychiatric conditions, many of which have been previously associated with transcriptional dysregulation. Understanding of factors that control striatal gene expression in mature tissues has paramount relevance to the pathology of disorders associated with these disorders.

Transcriptional regulation in the striatum has primarily focused on the development and differentiation of medium spiny neurons, which make up ∼90% of the neurons in the striatum. The transcription factor, B-cell leukemia/lymphoma 11B (Bcl11b) (a.k.a. chicken ovalbumin upstream promoter transcription factor interacting protein 2, CTIP2), which is predominantly expressed in striatal medium spiny neurons, plays an important role in striatal development [5]. However, we, and others, have demonstrated abundant striatal-enriched expression of Bcl11b in adulthood [5]–[7] (see Figure S1), suggesting that it plays important roles in controlling the expression of genes necessary to the functioning and maintenance of mature medium spiny neurons. Although Bcl11b has been shown to play critical roles in T-cell function and maintenance [8], its functional role in the adult brain is unknown.

Here we have used genome-scale chromatin immunoprecipitation followed by massively parallel sequencing (ChIP-Seq) in combination with genome-wide transcriptome analysis to identify and characterize Bcl11b target genes in striatal cells. Our findings reveal a large set of Bcl11b target genes in striatal cells, providing the first insight into core striatal gene regulation. We further identify a novel consensus binding motif for Bcl11b to account for direct Bcl11b-DNA binding. Importantly, we find that top Bcl11b target genes encode numerous components of the brain-derived neurotrophic factor (BDNF) signaling pathway. Altered BDNF signaling has been implicated in several neurodegenerative diseases, including Huntington's, Alzheimer's and Parkinson's diseases [9] and relevant therapeutic strategies for these disorders are aimed at increasing BDNF levels in the brain [10], [11]. We suggest that the design of small molecules to specifically interfere with Bcl11b-DNA interactions may represent a feasible therapeutic approach to elevate BDNF signaling.

## Results

### Striatal cell milieu

In order to identify candidate target genes for Bcl11b expression regulation, we analyzed transcriptome-wide expression profiles of wild-type, immortalized, striatal cells (ST*Hdh* striatal cells [12]) overexpressing Bcl11b. Because ST*Hdh* cells were derived from striata of embryonic day 14 tissue, we first tested whether wt ST*Hdh* striatal cells express markers of mature striatal neurons, as well as other striatal-enriched genes randomly selected from our previous studies [6], by standard PCR analysis. We find that ST*Hdh* cells express *Ppp1r1b*, encoding DARPP-32, the classic marker for mature medium spiny neurons, as well as mRNAs for other striatal markers, including neuronal guanine exchange factor (*Ngef*), brain-specific angiogenesis inhibitor 1-associated protein 2 (*Baiap2*), diacylglycerol kinase, epsilon (*Dgke*), regulator of G protein signaling 9 (*Rgs9*), copine V (*CpneV*), G protein, gamma 7 subunit (*Gng7*), adenylate cyclase 5 (*Adcy5*), Forkhead box protein P1 (*Foxp1*) and *Bcl11b* itself (Figure S2), suggesting that they share to some extent a similar molecular environment as in *vivo* medium spiny neurons.

### Identification of Bcl11b target genes by microarray analysis

**Figure 1:**
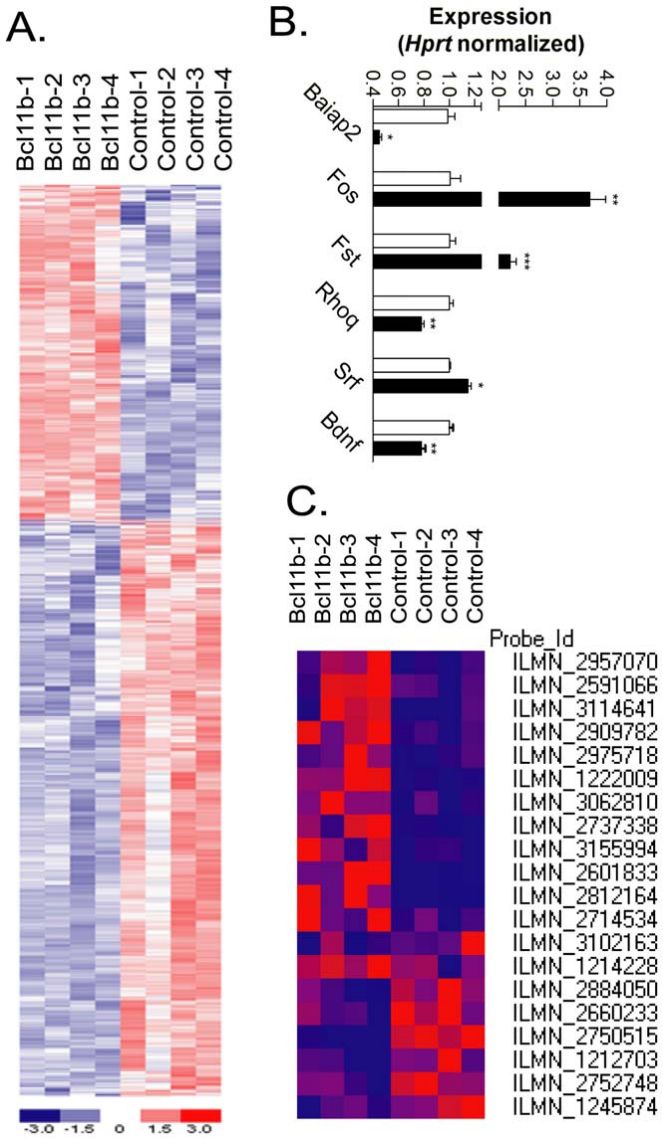
A. Heatmap depiction of the top 693 genes (FDR<0.10) differentially expressed in response to Bcl11b overexpression in striatal cells. Data are from n = 4 different sets of transfected cells. Heatmaps were generated with dChip program (www.dChip.org). Red indicates increased and blue indicates decreased expression relative to the mean transcript expression value. B. Real-time PCR validation of microarray expression differences elicited by Bcl11b in striatal cells. The genes are indicated by their official UniGene ID. *Hprt* was used to normalize gene expression differences. Open bars represent mock-transfected striatal cells, closed bars represent Bcl11b-transfected striatal cells. Asterisks denote significant differences in Bcl11b-transfected striatal cells compared to mock-transfected cell, as determined by ANOVA *, p<0.05; **, p<0.01; ***, p<0.001. C. Heatmap expression changes of genes related to the BDNF signaling pathway.

We performed microarray analysis using the Illumina Array MouseRef-8 v2 chips on Bcl11b- transfected striatal cells and found 694 genes (256 upregulated and 436 downregulated) whose expression was significantly altered by Bcl11b (at p<0.0027; FDR<0.10); these gene expression changes are depicted in Figure 1A. Complete lists of genes differentially expressed in Bcl11b- transfected striatal cells compared to untransfected cells are provided in Table S1. We validated the Bcl11b-induced expression changes of selected genes by real-time PCR analysis. These included FBJ osteosarcoma oncogene (*Fos*), Follistatin (*Fst*), Brain-specific angiogenesis inhibitor 1-associated protein 2 (*Baiap2*), Ras homolog gene family, member Q (*Rhoq*), Serum response factor (*Srf*) and Brain-derived neurotrophic factor (*Bdnf*) (Figure 1B).

Given that Bcl11b shows enriched expression in the striatum (see Figure S1), we searched for other striatal-enriched genes in our microarray datasets. From a list of 64 striatal-enriched genes, determined from our previous studies [6], nearly one-third (n = 18) were significantly altered in their expression in Bcl11b-transfected cells (p<0.05) (Table 1). This represents a statistically significant overrepresentation of striatal-enriched genes as Bcl11b targets compared to genes showing generalized expression throughout the brain (Fisher's Exact test; two-tailed; p<0.023). Notably, n = 58 previously designated striatal-enriched genes gave hybridization “Present” calls on our microarray chips, further indicating that these cells resemble the neuronal subtype from which they were derived.

**Table 1:** Striatal-enriched genes regulated by Bcl11b.

Gene ID:	Gene Description:	Fold-change:	P-Value:	FDR:	Acc #:
*Actn2*	Alpha-actinin 2	1.933	0.001	0.064	NM_033268
*Foxp1*	Forkhead box P1	1.370	0.033	0.284	AF339103
*Pde1b*	Phosphodiesterase 1B, Ca2+-calmodulin dependent	0.623	0.001	0.067	NM_008800
*Rgs9*	Regulator of G-protein signaling 9	1.478	0.043	0.476	NM_011268
*Rin1*	Ras and Rab interactor 1	0.654	0.001	0.047	NM_145495
*Arpp19*	cAMP-regulated phosphoprotein 19	0.751	0.012	0.190	NM_021548
*Baiap2*	BAI1-associated protein 2	0.601	0.001	0.071	NM_130862
*Bcl11b*	B-cell leukemia/lymphoma 11B	0.665	0.018	0.374	NM_021399
*Ensa*	Endosulfine alpha	1.374	0.003	0.105	NM_019561
*Foxp2*	Forkhead box P2	0.748	0.007	0.162	NM_053242
*Kcnip2*	Kv channel-interacting protein 2	0.657	0.036	0.296	NM_001034005
*Klf16*	Kruppel-like factor 16	1.590	0.007	0.148	NM_078477
*Ngef*	Neuronal guanine nucleotide exchange factor	0.752	0.005	0.131	NM_019867
*Osbpl9*	Oxysterol sterol binding protein 8	0.793	0.013	0.197	NM_1754589
*Pde10a*	Phosphodiesterase 10A	0.698	0.011	0.185	NM_011866
*Rap1gap*	Rap1 GTPase-activating protein	1.397	0.040	0.307	NM_001081155
*Rarb*	RAR beta receptor 1	0.552	0.006	0.140	NM_011243
*Zfp521*	Zinc finger protein 521	0.704	0.003	0.108	NM_145492

### ChIP-Seq analysis of Bcl11b binding sites

**Figure 2:**
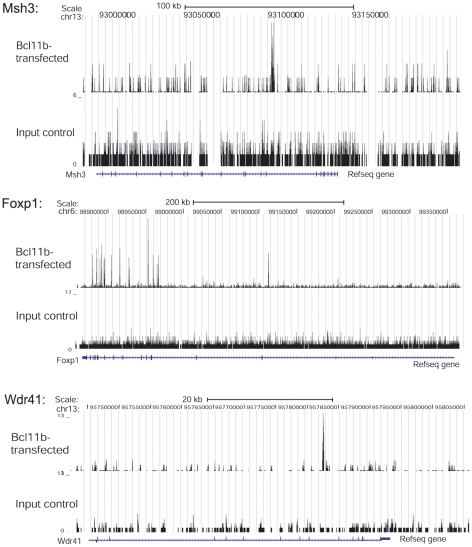
ChIP-seq binding patterns for Bcl11b target genes *Msh3*, *Foxp1* and *Wdr41*. The number of tags reflecting the ChIP enrichments is plotted on the y axis and chromosomal coordinates are shown on the x axis. RefSeq genes are in the orientation indicated by arrows on the gene representation. Enriched peaks have the following coordinates: Msh3 coordinates: chr13: 93085721–93085950; Foxp1 coordinates: chr6: 99112792–99113024; Wdr41 coordinates: chr13: 95782433–95782649.

In order to identify the genome-wide binding patterns of Bcl11b, we performed chromatin immunoprecipitation with a Bcl11b antibody in transfected striatal cells followed by massively parallel sequencing of the co-immunoprecipitated genomic DNA fragments (ChIP-Seq). We applied MACS software to identify Bcl11b-enriched binding regions. Examples of several target genes are shown in Figure 2. We then mapped the Bcl11b binding regions to genes using the UCSC Genome Browser and identified 1,410 Bcl11b-significantly-enriched binding regions ranging in size from 61–428 bp genome-wide. 232 of these genes were within 10 kb of an annotated transcriptional start site (TSS), however, only 36 of these mapped to within 1 kb of the TSS, suggesting that the majority of Bcl11b binding sites are far away from the proximal promoter regions. Further annotation of the top hits found that a majority of sequences within 10 kb of the TSS were within an intronic region (Table S2). We also observed that a Bcl11b ChIP peak overlaps with exon 1 of the Bcl11b gene itself, suggesting that, like many other eukaryotic transcription factors [13], Bcl11b regulates its own expression, as we have previously proposed [14].

**Figure 3:**
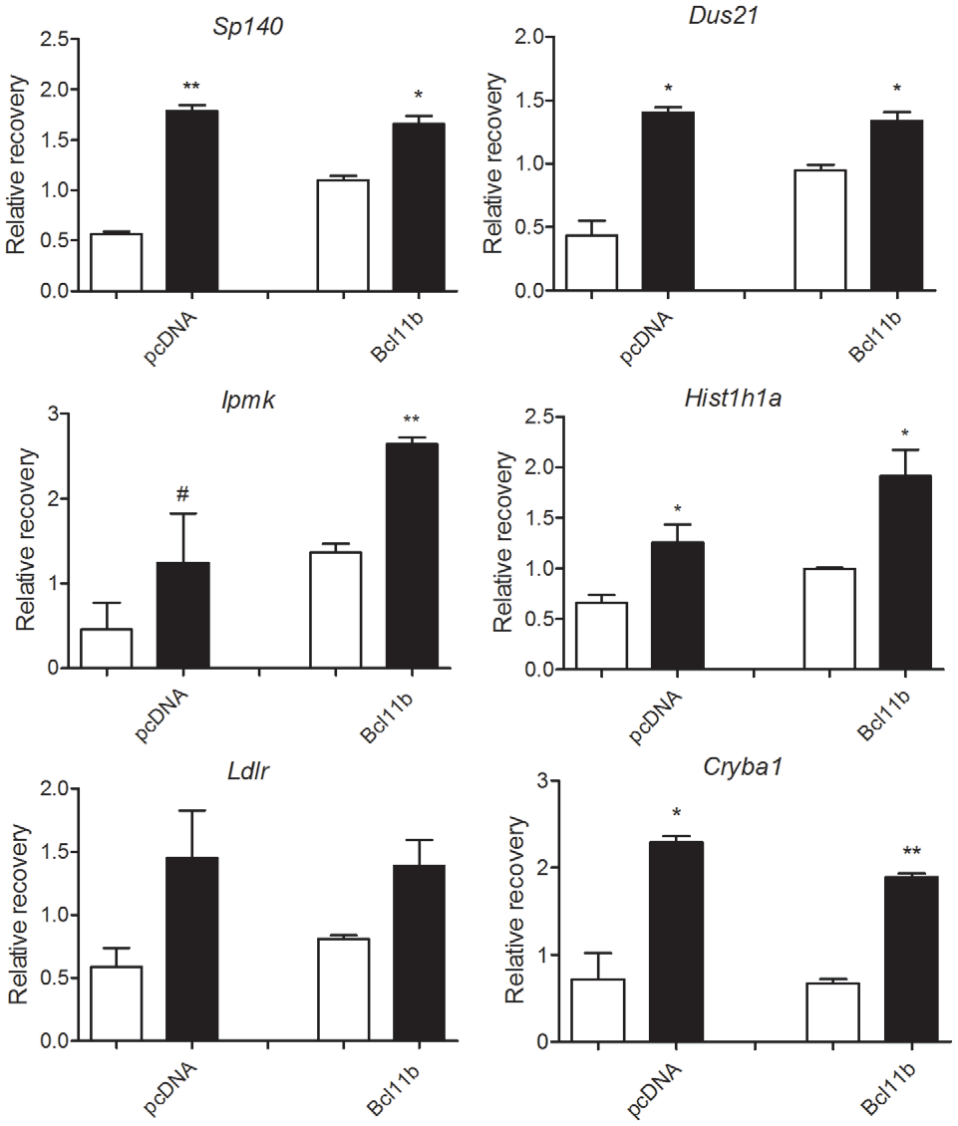
Chromatin-immunoprecipitation (ChIP)-qPCR for Bcl11b target genes. ChIP was performed using an anti-Bcl11b or control IgG antibody on both mock-transfected (pcDNA3.1) (open bars) and Bcl11b-transfected (closed bars) striatal cells as described in Methods. Real-time qPCR analysis was performed on the immunoprecipitated DNA using primers designed to amplify regulatory regions of the indicated genes.

We validated ChIP-seq peaks located near the TSSs of annotated genes using standard ChIP followed by real-time qPCR analysis. This analysis revealed enriched binding of Bcl11b (over IgG control) to the proximal promoter regions of several genes, including, Sp140 nuclear body protein (*Sp140*), Low density lipoprotein receptor (*Ldlr*), Dihydrouridine synthase 2-like (*Dus2l*), Histone cluster 1, H1a (*Hist1h1a*), Inositol polyphosphate multikinase (*Ipmk*), Crystallin, beta A1 (*Cryba1*) (Figure 3). Bcl11b binding to these genomic regions was also detected in un-transfected striatal cells, which endogenously express Bcl11b, albeit at low levels.

### Identification of consensus DNA-binding motifs and potential cofactors

**Figure 4:**
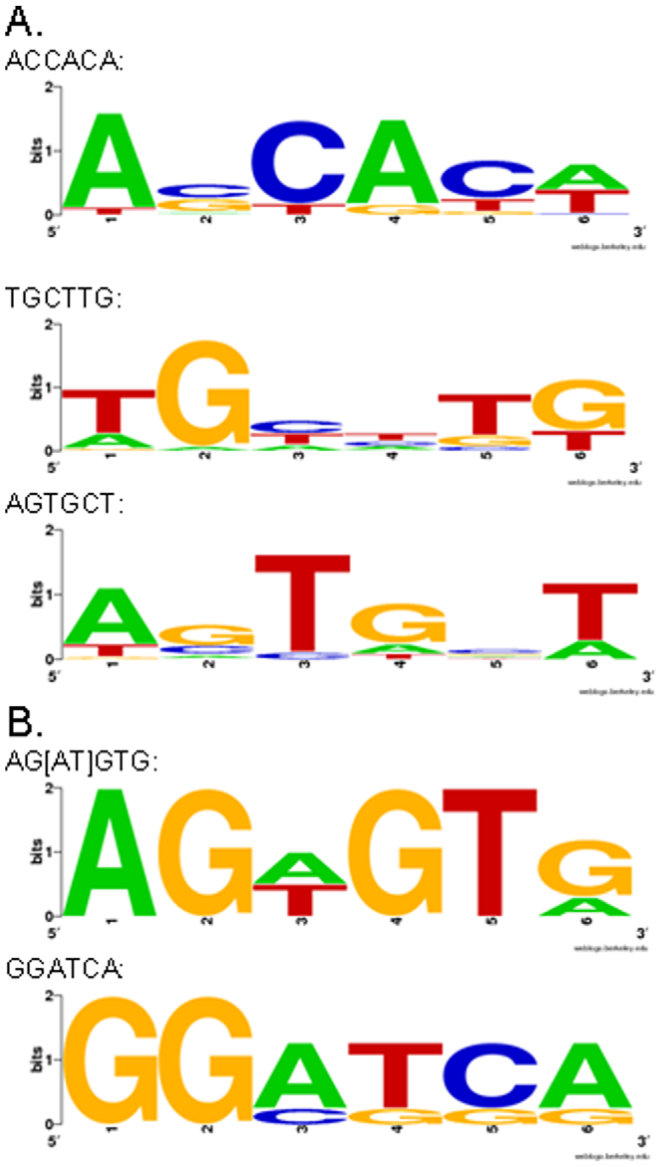
DNA binding consensus sequences for Bcl11b. A. Bcl11b binding motifs identified in all of the top ChIP-seq retrieved sequences. B. Bcl11b binding motifs in sequences from the 5′ regions.

We searched for consensus binding motifs for Bcl11b using MEME [15] and Motif Map [16] in the retrieved sequences of the Bcl11b binding regions. We found several potential consensus binding motifs and used the Bayesian Branch Length Score (BBLS) [16] to assess their evolutionary conservation in the multiple alignment of the mouse genomes with other genomes from the UCSC genome browser (29). The top consensus logos (BBLS score cutoff >1) identified in >25 separate Bcl11b binding sequences, regardless of genomic location, are ACCACA, TGCTTG and AGTGCT (Figure 4A). We also searched for consensus motifs in just the Bcl11b binding regions present in the annotated 5′ region of a given gene and found additional significant putative motifs, AG[AT]GTG and GGATCA (Figure 4B).

We next assessed whether other TFs could bind to the Bcl11b binding regions identified by ChIP-seq, potentially acting as Bcl11b cooperators for the regulation of gene expression. We searched for already annotated transcription factor motifs in mouse from the TRANSFAC database [17]. Several motifs for other factors, including AML1, MAFB, and HNF4, were found to have a significant BBLS score on subsets of the top ChIP-seq binding hits (Table 2). In addition, Bcl11b has been shown to alter transcription in HEK293 cells by interacting with chicken ovalbumin upstream promoter transcription factor (COUP-TF1; *Nr2f1*) [18]. Searching the same ChIP-seq hits for the previously defined COUP-TF 1 binding 6-mer (AGG/TTCA and TGTACT [19], revealed that 32.4% of the sequences contained one of these three sites.

**Table 2:** TRANSFAC mouse motifs.

Motif ID:	Motif Name:	Motif:	#:	Genes:
M01658	AML1	TGTGGTT	13	*Cdh23 Ddx28 FoxP1 Id3 Ipmk Mocos Msh3 Rundc3b Sh3rf3 Sp140 Tmx4 Trpm8 Wdr41*
M00751	AML1	TGTGGT	13	*Cdh23 Ddx28 FoxP1 Id3 Ipmk Mocos Msh3 Rundc3b Sh3rf3 Sp140 Tmx4 Trpm8 Wdr41*
M01227	MAFB	GCTGAC	12	*Cdh23 Cisd1 FoxP1 HIST1h1a Id3 Msh3 Rlf Sh3rf3 Sp110 Trpm8 Ubtd1 Wdr41*
M01033	HNF4	GGGGCA	11	*Bcl11b Cdh23 FoxP1 HIST1h4a Id3 Ipmk Odz2 Sh3rf3 Sp140 Ubtd1 Uhrf1bp1*
M01268	FXR	AGGTCAT	10	*Bcl11b Cdh23 Cisd1 FoxP1 HIST1h4a Ipmk Mocos Sh3rf3 Ubtd1 Uhrf1bp1*
M01181	Nkx3-2	TAAGTG	10	*Bcl11b Cdh23 Ddx28 FoxP1 Ipmk Msh3 Odz2 Rlf Sp140 Uhrf1bp1*
MA0078	Sox17	CCCATTGTC	9	*Adamts6 Cisd1 Ipmk Mocos Msh3 Sh3rf3 Tmx4 Trpm8 Ubtd1*
M01282	PPARA	TGACCTC	9	*Cdh23 Cisd1 FoxP1 Rapgef1 Sh3rf3 Trpm8 Ubtd1 Uhrf1bp1 Wdr41*
M01270	PPARG	AGGTCAG	9	*Bcl11b Cdh23 FoxP1 HIST1h4a Ipmk Mocos Msh3 Odz2 Sh3rf3*
M01104	MOVO-B	GCGGGGG	9	*Bcl11b Cdh23 Chd6 Cisd1 FoxP1 Id3 Sh3rf3 Ubtd1 Uhrf1bp1*
M01269	NURR1	TGGCCTT	8	*Cisd1 FoxP1 Id3 Ipmk Rapgef1 Sh3rf3 Trpm8 Uhrf1bp1*
M01032	HNF4	AGGTCA	8	*Bcl11b Cdh23 HIST1h4a Ipmk Odz2 Rlf Sh3rf3 Ubtd1*
M00921	GR	TCTGTTCT	8	*Bcl11b Dus2l Ipmk Mocos Msh3 Rapgef1 Rundc3b Wdr41*
M00240	Nkx2-5	TCAAGTG	8	*Bcl11b Chd6 Id3 Ipmk Odz2 Rapgef1 Sh3rf3 Uhrf1bp1*
M01709	MAFA	TCAGCAG	7	*FoxP1 HIST1h4a Id3 Mocos Msh3 Odz2 Sh3rf3*
M01118	WT1	CCCCCCCCC	7	*Cisd1 Id3 Ipmk Sh3rf3 Trpm8 Ubtd1 Wdr41*
M00726	USF2	CACGTG	7	*Bcl11b Cdh23 Chd6 Ddx28 Mocos Sh3rf3 Wdr41*
M00468	AP-2rep	CAGTGGG	7	*Chd6 FoxP1 Id3 Ipmk Mocos Msh3 Sh3rf3*
MA0122	Nkx3-2	CTAAGTGGC	6	*Bcl11b Cdh23 FoxP1 Msh3 Odz2 Uhrf1bp1*
MA0067	Pax2	AGTCACGC	6	*Bcl11b Chd6 Id3 Odz2 Tmx4 Ubtd1*
M01287	Neuro	CAGCTG	6	*Cdh23 FoxP1 Id3 Mocos Msh3 Sh3rf3*
M01243	MTF1	TGCGCAC	6	*FoxP1 Msh3 Sh3rf3 Ubtd1 Uhrf1bp1 Wdr41*
M00801	CREB	CGTCAC	6	*Chd6 HIST1h4a Lcor Mocos Sh3rf3 Ubtd1*
M00499	STAT5A	CACTTCTC	6	*Cisd1 Id3 Lcor Odz2 Sp140 Trpm8*
M01721	PUR1	GGGCCAGGG	5	*Cdh23 Chd6 FoxP1 Sh3rf3 Sp140*
M01694	YY2	CCATTAC	5	*Adamts6 Cdh23 Id3 Msh3 Wdr41*
M01665	IRF8	AGTTTCA	5	*HIST1h4a Ipmk Lcor Sh3rf3 Zfp474*
M00963	T3R	CCTGTCCTT	5	*Rapgef1 Sh3rf3 Trpm8 Ubtd1 Uhrf1bp1*
M00913	MYB	CAACTGCCC	5	*Cdh23 FoxP1 Ipmk Trpm8 Ubtd1*
M00712	myogenin	GGCAGCTG	5	*Cdh23 FoxP1 Id3 Msh3 Sh3rf3*
M00492	STAT1	CACTTCCG	5	*Adamts6 Ipmk Lcor Trpm8 Ubtd1*
M00272	p53	AGGCATGCCC	5	*Bcl11b FoxP1 Rapgef1 Sh3rf3 Ubtd1*
M00159	C/EBP	TGTGTGGTAAGGC	5	*FoxP1 Ipmk Mocos Sp140 Wdr41*

P-value cut-off<0.001 and BBLS cut-off>1.

### Integrated analysis of Bcl11b direct targets

To identify the most probable Bcl11b target genes, we compared the list of genes whose expression levels were altered by Bcl11b (at p<0.05) to the complete list of gene hits showing Bcl11b binding from the ChIP-seq assay. We obtained an integrated list of 247 top target genes for Bcl11b (Figure S3). Among them, 149 of the binding regions resulted in a downregulation of gene expression, while 98 were associated with an upregulation of gene expression (Figure S3). We asked whether examination of the set of integrated Bcl11b target genes could suggest potential functions for this TF in striatal neurons. We performed functional annotation of target genes using DAVID bioinformatics resources, which includes databases curating Gene Ontology (GO) terms, protein-protein interactions, protein functional domains, disease associations, and known pathways. To reduce the redundancy of common GO screening, we used the Functional Annotation Clustering tool, which displays similar annotations together. Several interesting annotation clusters were identified, including those related to calcium signaling, phosphorylation and regulation of small GTPase activity and phosphorylation (Table S3). We also find that Bcl11b target genes include several zinc finger–containing proteins of the C_2_H_2_ class, including *Brpf1*, *Klf8*, *Zfp41*, *Zfp428*, *Zfp 647*, *Bcl6b*, *Hivep2*, *Zbtb17*, *Trerf1*, *Snai1*, *Zbtb4*, *Foxp1* and *Bcl11b* itself (Table S3).

**Figure 5:**
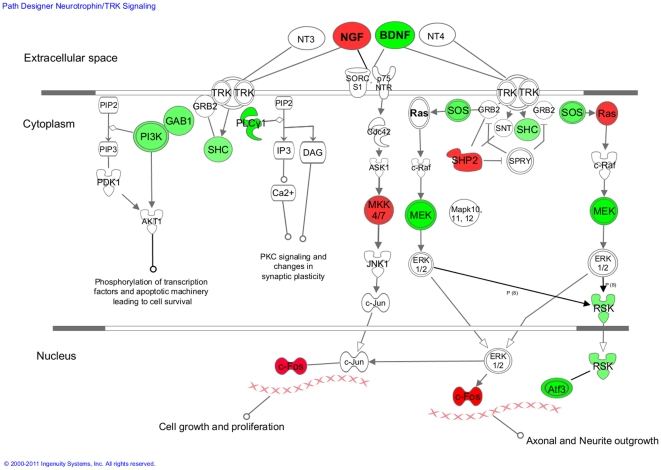
Schematic depiction of the neurotrophin signaling pathway. Those gene indicated in red represent genes upregulated in expression and those in green, downregulated in expression, in response to Bcl11b. Figure generated using Pathway Design by Ingenuity Systems Pathways Analysis. Abbreviations for the official gene symbols as follows: BDNF, *Bdnf*; NGF, *Ngfb*; PI3K, *Pik3r1*; GAB1, *Gabl1*; SHC, *Shc1*; PLCγ1, *Plcg2*; Ras, *Kras*; SOS, *Sos1*; SHP2, *Ptpn11*; MKK4/7, *Map2k7*; MEK, *Map2k1*; RSK, *Rps6ka1*; c-fos, *Fos*; Atf3, *Atf3*.

We next focused on the KEGG and PANTHER pathways of the DAVID database. A critical pathway downstream from Bcl11b in the striatum was revealed to be BDNF/neurotrophin signaling, with seven genes making up this pathway appearing as integrated Bcl11b targets. These include *Rps6ka5*, *Irak4*, *Plcg2*, *Mapk9*, *Shc1*, *Mapk10* and *Rapgef1* (Table S4; Figure 5). The ErbB, or Epidermal Growth Factor (EGF), signaling pathway also was significantly associated with Bcl11b, with six target genes appearing in this pathway: *Nrg3*, *Pak4*, *Plcg2*, *Mapk9*, *Shc1*, and *Mapk10* (Table S4). To expand the pathways analysis, we used the entire list of Bcl11b-induced gene expression changes as input and found that n = 20 genes related to neurotrophin signaling were altered in expression by Bcl11b, a majority of which were downregulated in expression (Figures 1C and Figure 5). Taken together, these data reveal that Bcl11b plays an important role in growth factor signaling in striatal cells, primarily acting as a negative regulator of this pathway.

## Discussion

Precise regulation of gene expression in the striatum is essential to its specific functions, which include the planning and control of movement and motor function, as well as a variety of other cognitive processes. Transcriptional dysregulation in striatal cells is associated with severe movement disorders and psychiatric conditions. Hence, a better understanding of the mechanisms of gene expression regulation in adult striatum is essential. The transcription factor, Bcl11b, is a C_2_H_2_ zinc finger protein, which exhibits enriched expression in the striatum; thus we have hypothesized that it is important in the control of striatal gene expression, although its exact functional role has remained unknown. In this study, we used chromatin immunoprecipitation followed by high-throughput sequencing (ChIP-Seq) in combination with transcriptome-wide expression profiling, to identify a large, unbiased set of Bcl11b target genes. We integrated the list of genes whose expression levels were altered by Bcl11b to the complete list of gene hits showing Bcl11b binding from the ChIP-seq assay, obtaining a list of 247 direct target genes for Bcl11b regulation. This list contains genes both up- and down-regulated expression in response to Bcl11b. We validated these high-throughput findings by independent ChIP-qPCR and real-time PCR expression assays, providing confidence in our global output of Bcl11b target genes.

Subjecting our integrated Bcl11b gene target list to pathway analysis, we find that Bcl11b regulates numerous genes of the BDNF signaling pathway. Neurotrophins are a family of trophic factors involved in the development, differentiation and survival of CNS cells. The neurotrophin family consists of four members: nerve growth factor (NGF), brain derived neurotrophic factor (BDNF), neurotrophin 3 (NT-3), and neurotrophin 4 (NT-4) (Figure 5) of which BDNF is the most widely recognized. Neurotrophins exert their functions via activation of Trk tyrosine kinase receptors, which are linked to various intracellular signaling cascades, including the MAPK, PI-3 kinase and the PLC pathways. These signals play an important role for neural development and additional higher-order activities such as learning and memory. Accordingly, altered activities of BDNF and other neurotrophins have been implicated in several neurodegenerative diseases, including Huntington's, Alzheimer's and Parkinson's diseases [9]. Therapeutic strategies aimed at increasing BDNF levels in the brain are thought to be feasible treatment approaches in the clinic [10], [11]. We found that Bcl11b regulates the expression of genes encoding a wide array of components of the BDNF/neurotrophin signaling pathways, including *Bdnf* itself in cultured striatal cells. A majority of expression changes were all decreases, suggesting that Bcl11b is a negative regulator of BDNF signaling.

While we identified 247 genes showing overlap between our microarray and ChIP-seq datasets, this number represents only a fraction of the total genes showing altered expression by Bcl11b from the microarray dataset. Many of these expression alterations are of great interest. For example, we found that nearly one-third (n = 18) of previously identified striatal-enriched genes were significantly altered in their expression in Bcl11b-transfected cells, indicating that Bcl11b is a central regulator of striatal-specific gene expression. However, only one of these contained a Bcl11b binding region, as determined by ChIP. Several reasons could account for this apparent discrepancy. One possibility is that some genes identified in the microarray study represent secondary effects of primary gene expression changes elicited directly by Bcl11b. We found that Bcl11b regulates the levels of several other TFs; hence, it is possible that differentially expressed genes are targets of one of these other factors. Second, it is possible that the expression regulation of some of these genes requires a cofactor, and that protein-protein interactions of Bcl1b with a cofactor mask the pull-down of the chromatin immunoprecipitation step. For example, Bcl11b has been shown to alter transcription in HEK293 cells by interacting with COUP-TF family members, in particular COUP-TF1 [18] and we found that several of the ChIP sequences contained a known consensus binding domain for COUP-TF1. Finally, ChIP assays have technical limitations, such as the sensitivity of the antibody, and they may not detect transient protein-DNA interactions.

We found a total of 1,465 Bcl11b-binding regions in the mouse genome, however, only 232 of these mapped to within 10 kb of a TSS and only 36 of these within 1 kb. This suggests that Bcl11b acts distally, rather than acting at the proximal promoter regions of genes. Bcl11b has previously been shown to bind a CTIP response element that is related to the canonical GC box [20], although this motif, GGCCG/AG/AAGG, was not present in any Bcl11b binding regions in striatal cells. However, we did identify several novel Bcl11b binding motifs, including ACCACA, TGCTTG and AGTGCT. Additional significant motifs, AG[AT]GTG and GGATCA, were also identified when searching Bcl11b-binding sequences in the 5′ regions of the genes. A recent study examining the DNA binding domains of a large group of TFs revealed that ∼half of the TFs recognized multiple, distinct sequence motifs, indicating flexibility in TF binding recognition [21].

The identification of a specific Bcl11b binding motif has potential therapeutic relevance, as it is possible to design small molecules that target specific DNA sequences [22], [23]. Synthetic polyamides containing N-methylimidazole and N-methylpyrrole amino acids show high affinity and specificity for predetermined DNA sequences. In particular, previous studies have shown that an eight-ring polyamide targeted to a specific region of the transcription factor TFIIIA binding site can interfere with 5S RNA gene expression in Xenopus kidney cells [22]. Identification of the relevant DNA motif for Bcl11b binding to promoter regions of genes in the BDNF signaling pathway could lead to the design of similar molecules, which would represent a novel therapeutic approach to up-regulating the BDNF pathway in striatal neurons. Such a therapeutic approach would be applicable to several neurodegenerative diseases, including Huntington's, Alzheimer's and Parkinson's diseases, in which decreased BDNF signaling has been implicated [9].

One cautionary note when translating our findings into in vivo striatum gene regulation is that the striatal cells used in this study were immortalized from embryonic day 14 tissue. Although we show that these cells express DARPP-32, a marker of mature medium spiny neurons [24], as well as several other genes that are selectively expressed in the adult striatum, it is possible that the global gene expression profiles in these cells are different compared to *in vivo* medium spiny neurons.

In summary, these findings represent the first comprehensive genome-wide study of Bcl11b binding, and coupled with transcriptome profiling, reveal important roles for Bcl11b in the regulation of striatal gene expression and function. This approach allows for the identification of potential disease-related pathways under the controls of Bcl11b and accordingly, these findings illuminate neurotrophin signaling pathway as a primary target pathway for Bcl11b regulation. Therapeutic strategies aimed at increasing BDNF levels in the brain are thought to be feasible treatment approaches for several neurodegenerative disorders. Our findings suggest that specific targeting of the Bcl11b/DNA interaction represents a novel means to upregulate the BDNF pathway. Importantly, due to the striatal-enriched expression of Bcl11b, targeting this TF would not result in widespread effects in other brain regions.

## Materials and Methods

### Striatal Cell Culture and RNA Preparation

Conditionally immortalized wild-type ST*Hdh^Q7^*striatal neuronal progenitor cells were a kind donation from Dr. Marcy MacDonald [12]. The striatal cells were grown at 33°C in Dulbecco's modified Eagle's medium (DMEM) supplemented with 10% fetal bovine serum (FBS), 1% non-essential amino acids, 2 mM L-glutamine and 400 µg/ml G418 (Sigma-Aldrich, St Louis, MO). Cells were plated at 3×10^5^ cells per well in six well tissue culture plates. The following day the cells were transfected with an expression plasmid (pcDNA3.1) containing the cDNA of mouse Bcl11b (beta isoform; nts 272–2710 of accession # AB043553) using Polyjet transfection reagent (SignaGen, Ijamsville, MD) according to the manufacturer's instructions. Empty expression vector, pcDNA3.1, was used to ensure all wells received the same amount of total DNA. Transfection efficiency was assessed in duplicate sets of transfected cells by quantifying the percentage of green fluorescent protein (GFP)-positive cells using fluorescence microscopy and by measuring Bcl11b mRNA expression with qPCR. Two days after transfection, cells were harvested, and RNA was extracted using RNAeasy mini kit (Qiagen) with DNase I treatment to eliminate genomic DNA contamination.

### Microarray Experiments

Total RNA was quantified using NanoDrop (ND-1000, manufacturer) and quality was checked with the Agilent 2100 Bioanalyzer using the RNA 6000 Nano LabChip. One microgram (1 µg) of total RNA was taken through Ambion's Illumina TotalPrep RNA Amplification System (protocol available at http://ambion.com/techlib/prot/fm_IL1791.pdf). Post amplification RNA (750 ng cRNA) product was hybridized onto the Illumina Sentrix BeadChip Array MouseRef-8 v2 for 18 hours at 58°C (protocol available at http://www.illumina.com/products/mouseref-8_expression_beadchip_kits_v2.ilmn). After hybridization, the BeadChip Arrays were washed and stained as per protocol requirements. BeadChip Arrays were scanned using the Illumina BeadArray Reader with default settings. Data normalization was performed using GenomeStudio Gene Expression Module v1.0 with quantile normalization [25], [26]. The sample clustering was done using BRB-ArrayTools (http://linus.nci.nih.gov/BRB-ArrayTools.html), with centered correlation and average linkage. The Limma package in the R software was used to find transcripts showing differential expression in response to Bcl11b overexpression [27]. This microarray data has been deposited in the NCBI GEO database with Accession # GSE31096. Results are presented as a fold change in expression level with an absolute 1.25-fold change cut-off, and p-value<0.05 (see Table S1).

### Chromatin Immunoprecipitation (ChIP)

ChIP assays on cultured striatal cells were performed as described in previous studies [28]. Briefly, cellular homogenates were cross-linked by incubating with 1% of formaldehyde for 15 min at room temperature, and then homogenizing to isolate nuclei. DNA was to achieve ∼0.2–1.0 kb sized DNA fragments. An aliquot of precleared homogenates was incubated with 2 µg of rabbit anti-Bcl11b antibody (Bethyl Laboratories, Montgomery, TX) or 3 µg of control IgG (BD Biosciences) overnight at 4°C, followed by hybridization to 60 µl of protein-sepharose A beads. The protein-DNA complexes were washed on the beads, elution buffer added and the eluate incubated with proteinase K to digest protein. The cross-linking reaction was reversed by incubating at 65°C overnight and DNA was purified with DNA purification kit (ZYMO research Corp). The recovered pull-down DNA, as well as input DNA, was used for massively parallel sequencing (ChIP-seq below) or quantified by real-time PCR analysis for ChIP-PCR.

### Chromatin-immunoprecipitation-sequencing (ChIP-seq)

ChIP-seq sequencing libraries were prepared using 10 ng of DNA prepared by ChIP above (pull-down and input). ChIP DNA samples ends were repaired and DNA products were purified using DNA Clean&Concentrator™-5 Kit (Zymo Research, Irvine, CA). Next, DNA ends were A-tailed and again purified using the DNA Clean&Concentrator™-5 Kit. Illumina Paired End-adapter oligonucleotides (0.33 µM) were then ligated to the A-tailed cDNA ends and purified as before. The DNA library products were separated on an Invitrogen 2% Size-Select agarose gels and products corresponding to a size of approximately 200–250 bases were removed from the gel and cleaned using the Agencourt SPRI system. The DNA material was PCR amplified and purified on 2% NuSieve GTG® agarose gel, excised, and isolated again using Zymoclean™Gel DNA recovery kit. The purified DNA library was quantified using the Qubit quantification platform (Invitrogen, Carlsbad, CA) and sized using the 2100 Bioanalyzer. DNA products were then denatured in 0.1 N NaOH and diluted to a final concentration of 10 pM before being loaded onto the Illumina paired-end flow-cell for massively parallel sequencing by synthesis on the Illumina GAIIx.

The Genome Analyzer Pipeline Software v1.7 was used to perform the early data analysis of a sequencing run, including the image analysis, base calling, and alignment. Alignment was performed with Efficient Large-Scale Alignment of Nucleotide Databases (ELAND2). The uniquely aligned reads containing less than 3 errors to the mouse genome are used as input to the Model-based Analysis for ChIP-Seq (MACS) program, a publicly available open source ChIP-Seq analysis (http://liulab.dfci.harvard.edu/MACS/) [29]. Peaks found by MACS (v1.4alpha2) were mapped to within 10 kb of a RefSeq transcript of the mouse version mm9 database. The peak coordinates were then positioned outside the 5′ or 3′ transcript coordinates, or within exons/introns coordinates for the annotated genes, by comparing to the transcript and exon start/end positions from the UCSC Genome Browser. The peak sequences were obtained from queries to the NCBI Entrez database using the chromosome coordinates.

### DNA Motif Analysis

*De novo* motif finding was performed using MEME [15] on all of the top ChIP-seq binding hits for Bcl11b (Table S3) and on the subset of hits from the 5′ region. On the two sets, MEME was run both with default parameters and with the minimum number of targets for each motif equal to the number of sequences in the respective set. For each run, the top 5 motifs were retained, obtaining a total of 20 putative motifs. MotifMap [16] was used to filter this set of putative motifs by comparative genomic conservation. The current version of MotifMap for the mouse genome uses the multiple alignment of 30 model organisms' genomes, downloaded from the UCSC genome browser [30], and the corresponding phylogenetic tree to score the degree of conservation of putative transcription factor binding sites using the Bayesian Branch Length Score (BBLS).

A BBLS score >1 denotes a significant level of conservation across a non-trivial subset of organisms in the reference tree; values >2 denote a high level of conservation. The putative motifs detected with MEME were filtered by selecting only those for which at least four distinct ChIP-seq hits obtained a BBLS score >1.

Detection of already annotated motifs was performed using all (over 800) mouse transcription factors in the TRANSFAC 11.3 database [17]. The log-odd TRANSFAC motif matrices were used to select those regions in the ChIP-seq hits with a Z-score p-value less than 0.001. The background mean and standard deviation for each motif-matching score were obtained by random sampling 10 millions locations in non-repeat regions of the mouse genome. As above, each single motif-match was validated with MotifMap and only those motifs for which at least four ChIP-seq hits obtained a BBLS score >1 were retained (Table 2).

### Real-Time PCR Analysis

Real-time PCR analysis was performed on the recovered DNA from the ChIP experiments using primers directed against the promoter regions of striatal-enriched genes (Table S5) or on cDNA prepared from striatal cells using the primers designed in the exonic regions of selected genes (Table S5) as described previously [6], [31]. Primers were designed to generate amplicons of 80–150 nucleotides with similar melting temperatures (64°C) using Invitrogen's Primer Designer.

## Supporting Information

Figure S1 **CNS Expression of Bcl11b.** *In situ* hybridization analysis was performed on free-floating coronal (A–F) and sagittal sections (G) (25 µm-thick) from wild type C57black6J mice (2 months of age). Antisense ^35^S-labeled riboprobes directed against Bcl11b were hybridized to brain sections as described previously (Desplats et al., 2008). CPu, caudate putamen; Cx, cortex; Hipp, hippocampus; Thal, thalamus; Cb, cerebellum; OT, olfactory tubercle.
(PDF)

Figure S2 **ST** ***Hdh*** **striatal cells express several markers of mature medium spiny neurons, as indicated by their official UniGene IDs below.** Each gene was amplified from cDNA prepared from RNA from duplicate wells of ST*Hdh* striatal cells using standard PCR conditions.
(PDF)

Figure S3 **Venn diagram showing overlap of Bcl11b microarray gene expression and ChIP-seq data.** Gene expression changes at p<0.05 were used for the Venn overlaps.
(PDF)

Table S1 **List of genes differentially expressed in Bcl11b-transfected **ST***Hdh* striatal cells compared to mock (pcDNA3.1)-transfected cells.**
(XLSX)

Table S2 **Annotated ChIP-seq binding hits for Bcl11b in ST** ***Hdh*** **striatal cells.**
(XLSX)

Table S3 **Functional Annotation Clustering analysis of the integrated Bcl11b target genes (those appearing in both microarray and ChIP-seq datasets) using The Database for Annotation, Visualization and Integrated Discovery (** ***DAVID*** **) software v6.7.** The p-values associated with each annotation are according to the Fisher Exact/EASE Score.
(XLSX)

Table S4 **Pathways analysis of integrated Bcl11b target genes (those appearing in both microarray and ChIP-seq datasets) using The Database for Annotation, Visualization and Integrated Discovery (** ***DAVID*** **) v6.7 software, focusing on PANTHER and KEGG databases.** The p-values reflect the threshold of EASE Score, a modified Fisher Exact P-Value, for gene-enrichment analysis.
(XLSX)

Table S5 **Primers used for quantitative real-time PCR analysis and for ChIP-qPCR analysis.** The ChIP-qPCR primers show the position relative to the transcription start sites of the indicated genes.
(DOC)
