# Supplementary material for: Genome-Wide Identification of Bcl11b Gene Targets Reveals Role in Brain-Derived Neurotrophic Factor Signaling
Source: PLoS One. 2011 Sep 1;6(9):e23691. doi: 10.1371/journal.pone.0023691 (PMC3164671; doi:10.1371/journal.pone.0023691)

Supplementary Figure 1. CNS Expression of Bcl11b. *In situ* hybridization analysis was performed on free-floating coronal (A-F) and sagittal sections (G) (25 µm-thick) from wild type C57black6J mice (2 months of age). Antisense ^{35}S-labeled riboprobes directed against Bcl11b were hybridized to brain sections as described previously (Desplats et al., 2008). CPu, caudate putamen; Cx, cortex; Hipp, hippocampus; Thal, thalamus; Cb, cerebellum; OT, olfactory tubercle.

Bcl11b mRNA:

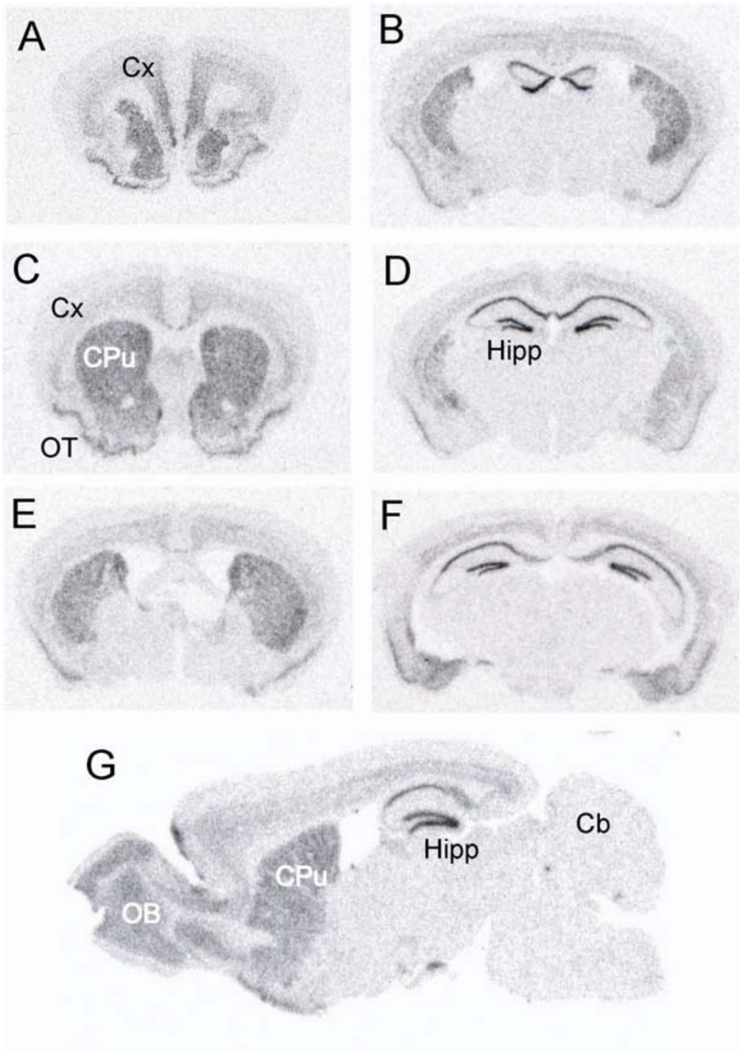

Supplement: Figure S1 — CNS Expression of Bcl11b. In situ hybridization analysis was performed on free-floating coronal (A–F) and sagittal sections (G) (25 µm-thick) from wild type C57black6J mice (2 months of age). Antisense 35S-labeled riboprobes directed against Bcl11b were hybridized to brain sections as described previously (Desplats et al., 2008). CPu, caudate putamen; Cx, cortex; Hipp, hippocampus; Thal, thalamus; Cb, cerebellum; OT, olfactory tubercle. (PDF) [file pone.0023691.s001.pdf]
