# Supplementary material for: Genome-Wide Identification of Bcl11b Gene Targets Reveals Role in Brain-Derived Neurotrophic Factor Signaling
Source: PLoS One. 2011 Sep 1;6(9):e23691. doi: 10.1371/journal.pone.0023691 (PMC3164671; doi:10.1371/journal.pone.0023691)

Supp. Figure 2. ST*Hdh* striatal cells express several markers of mature medium spiny neurons, as indicated by their official UniGene IDs below. Each gene was amplified from cDNA prepared from RNA from duplicate wells of ST*Hdh* striatal cells using standard PCR conditions.

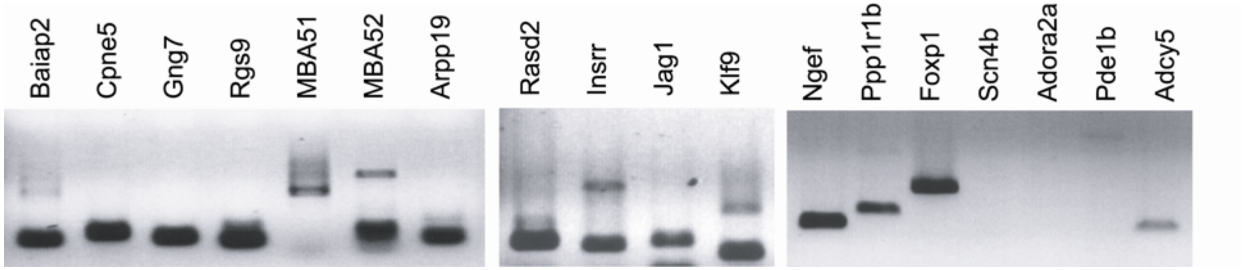

Supplement: Figure S2 — ST Hdh striatal cells express several markers of mature medium spiny neurons, as indicated by their official UniGene IDs below. Each gene was amplified from cDNA prepared from RNA from duplicate wells of STHdh striatal cells using standard PCR conditions. (PDF) [file pone.0023691.s002.pdf]
