# Supplementary material for: Genome-Wide Identification of Bcl11b Gene Targets Reveals Role in Brain-Derived Neurotrophic Factor Signaling
Source: PLoS One. 2011 Sep 1;6(9):e23691. doi: 10.1371/journal.pone.0023691 (PMC3164671; doi:10.1371/journal.pone.0023691)

Suppl Figure 3. Venn diagram showing overlap of Bcl11b microarray gene expression and ChIP-seq data. Gene expression changes at $p<0.05$ were used for the Venn overlaps.

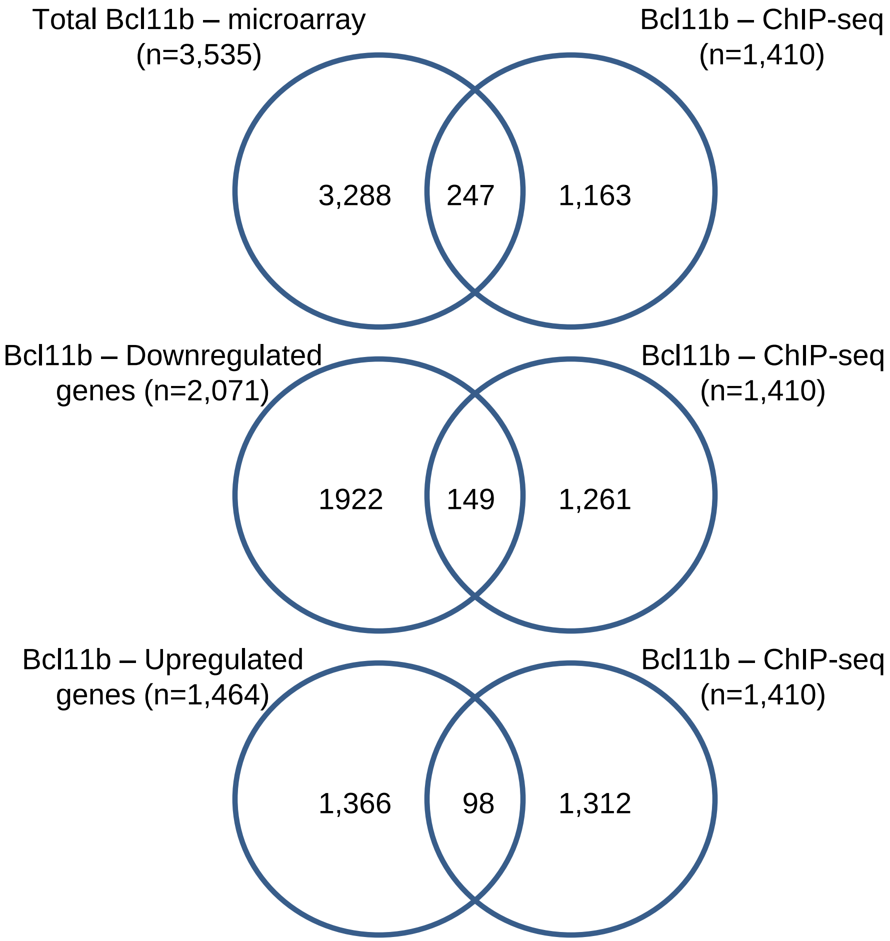

Supplement: Figure S3 — Venn diagram showing overlap of Bcl11b microarray gene expression and ChIP-seq data. Gene expression changes at p<0.05 were used for the Venn overlaps. (PDF) [file pone.0023691.s003.pdf]
