# Supplementary material for: Genome-Wide Identification of Bcl11b Gene Targets Reveals Role in Brain-Derived Neurotrophic Factor Signaling
Source: PLoS One. 2011 Sep 1;6(9):e23691. doi: 10.1371/journal.pone.0023691 (PMC3164671; doi:10.1371/journal.pone.0023691)
Supplement: Table S5 — Primers used for quantitative real-time PCR analysis and for ChIP-qPCR analysis. The ChIP-qPCR primers show the position relative to the transcription start sites of the indicated genes. (DOC) [file pone.0023691.s008.doc]

**Suppl. Table 5.**

**Primers used for quantitative real-time PCR analysis.**

Gene ID: Transcript ID #: Forward primer (5’- 3’): Reverse primer (5’- 3’):

Bcl11b NM_001079883 gaaaggcatctgtcccaagca gcacgcagcggtgaagtaatc

Foxp1 NM_053202 gcaggcggtactcagagaaa cactgtccatagtgccccttt

Baiap2 NM_001037755 gctgtagaatgtctctgtctcg acgcgtcgactcacactgtgg

C1ql1 NM_011795 caattgcccaggatgcagac gctttgcctccatccagctt

Fos NM_010234 cggcagaaggggcaaagtag tcagctccctcctccgattc

Fst NM_008046 **cttgctgggcagatccattg cagcgacctctgccaacctt**

Rhoq NM_145491 tctccgagatgaccccaaaact cactccacatagcagcatgcac

Hprt NM_012556 tgatgaaggagatgggaggcca ccagcaggtcagcaaagaacttatagc

Yyl NM_009537 gtggcaaagcgttcgttgag ctccggtatggattcgcaca

Srf NM_020493 tggtgtccctcaggtgttcc ggcatccaggttcaccacct

Rab18 NM_181070 tgaaatttgcacgcaagcat ctttcccacaggccaggtgt

Hmgcll1 NM_173731 ctccaggggctcttgctgtt ttgcataagggcagccacct

Eif3h NM_080635 gtatcagcagcgtcgccaac aatctggcctgcaatgagca

**Primers used for ChIP-qPCR and position relative to the transcription start sites**

**of the indicated genes.**

Gene: Transcript ID #: Primer sequences (5’- 3’): Position^a^

*Cryba1* NM_009965

Primer-1 Top: cagccaatagggcaagcaca -9,117 to -8,980

Primer-2 Top: agccccattgtcctctccac -3,670 to -3,525

Bottom: catgccagggatggttgcta

*Ipmk* NM_027184

Primer-1 Top: cagcagacacacaccctgga -9,316 to -9,170

Bottom: tgaggctaaagcacacacttgg

Primer-2 Top: tgcgcatgatcagtgtggag -308 to -206

Bottom: agcatgcgcaacagctactc

*Hist1h1a* NM_030609

Primer-1 Top: cgtagatgaggccggagatg -2,716 to -2,608

Bottom: agcgccaccgtaaggttctc

Primer-2 Top: gcgcaggtcggtcttgaagt -1,615 to -1,514

Bottom: agatccggcgctaccagaag

*Dus2l* NM_025518 Top: tgacgccctcttctggtctg -7,762 to -7,660

Bottom: tggttgtgagccttcatgtgg

*Sp140* NM_001013817 Top: agctactttgggggctgctg -6,640 to –6,539

Bottom: ctctccttccaccctccaga

*Ldlr* NM_010700 Top: tccagcccatcatctctgttg -6,427 to –6,531

Bottom: tcttgagtctgaggccatcca

^a^ The number represents the nucleotide position from ATG.
